# Understanding patient and family utilisation of community-based palliative care services out-of-hours: Additional analysis of systematic review evidence using narrative synthesis

**DOI:** 10.1371/journal.pone.0296405

**Published:** 2024-02-21

**Authors:** Joanna Goodrich, Caleb Watson, Inez Gaczkowska, Richard Harding, Catherine Evans, Alice Firth, Fliss E.M. Murtagh

**Affiliations:** 1 Cicely Saunders Institute of Palliative Care and Rehabilitation, King’s College London, London, United Kingdom; 2 King’s College London Medical School, London, United Kingdom; 3 Sussex Community NHS Foundation Trust, Brighton, United Kingdom; 4 Wolfson Palliative Care Research Centre, Hull York Medical School, University of Hull, Hull, United Kingdom; University of Sri Jayewardenepura, SRI LANKA

## Abstract

**Background:**

Community-based out-of-hours services are an integral component of end-of-life care. However, there is little understanding of how patients and families utilise these services. This additional analysis of a systematic review aims to understand and identify patterns of out-of-hours service use and produce recommendations for future service design.

**Method:**

Data on service use was extracted and secondary analysis undertaken, from a systematic review of models of community out-of-hours services. Narrative synthesis was completed, addressing four specific aspects of service use: 1.Times when patients/families/healthcare professionals need to contact out-of-hours services; 2. Who contacts out-of-hours services; 3. Whether a telephone call, centre visit or home visit is provided; 4. Who responds to out-of-hours calls.

**Results:**

Community-based out-of-hours palliative care services were most often accessed between 5pm and midnight, especially on weekdays (with reports of 69% of all calls being made out-of-hours). Family members and carers were the most frequent callers to of the services (making between 60% and 80% of all calls). The type of contact (telephone, centre visit or home visit) varied based on what was offered and on patient need. Over half of services were led by a single discipline (nurse).

**Conclusions:**

Out-of-hours services are highly used up to midnight, and particularly by patients’ family and carers. Recommendations to commissioners and service providers are to:

• Increase provision of out-of-hours services between 5pm and midnight to reflect the increased use at these times.

• Ensure that family and carers are provided with clear contact details for out-of-hours support.

• Ensure patient records can be easily accessed by health professionals responding to calls, making the triage process easier.

• Listen to patients, family and carers in the design of out-of-hours services, including telephone services.

• Collect data systematically on out-of-hours-service use and on outcomes for patients who use the service.

## Background

‘Out-of-hours’ services provide urgent care outside normal working hours, typically evenings (often 6pm-8am), weekends (from Friday evening to Monday morning), and public holidays [1]. Out-of-hours services are crucial for patients at the end-of-life with serious or life-limiting illnesses who may experience new or worsening symptoms at any time [2]. Patients may require out-of-hours care and support urgently, and if unavailable from community services, may attend an emergency department to obtain the healthcare needed. This can be distressing for the patient and their family [3, 4]. The need for community out-of-hours services is increasing with a growing ageing population and the resulting higher prevalence and burden of chronic conditions [5].

Patients near the end of life largely prefer to receive care in their usual residence, at home, and to die at home with the support and care in place [6]. Patients value being in a place where they are surrounded by family and friends, in a comfortable and familiar environment, where they can still have security and autonomy and they can die with dignity [7]. The goal of community-based palliative care services is to provide the best possible quality of life for patients and their families facing challenges associated with life-limiting illness, whether physical, psychosocial or spiritual [8]. These services are an integral component of end-of-life care. They can be divided into care provided by specialist palliative care (SPC) professionals, and care provided by generalists (professionals who do not specialise in palliative care) [10, 11]. Specialist palliative care is provided by healthcare professionals such as consultants in palliative medicine and clinical nurse specialists who have undergone specific palliative care training, undertake this role for most of their professional time, and have additional skills and experience to support and care for patients with complex needs as a result of their terminal illness. Care provided by those who are not SPC professionals (generalists) is provided by general practitioners and district nurses with general skills and experience in palliative care but for whom palliative and end-of-life care is not the main part of their work [9–12].

The provision of out-of-hours palliative care services in the community is highly variable in quality, and provision is inequitable [13, 14]. Out-of-hours services provide care for almost two-thirds of the week, but typically receive fewer resources than in-hours services [1, 15]. This is particularly evident in more rural and remote areas where there is poor availability of healthcare resources due to large geographical areas, workforce shortages, and limited infrastructure [16, 17].

These inequities have been highlighted for some time [16–19]. A recent review by Marie Curie amplified the inconsistent provision of care, particularly in deprived and more rural areas and for patients with non-cancer diagnoses [4, 18, 19]. Furthermore, out-of-hours palliative care services pose added challenges regarding patient safety. Challenges include care being delivered by several different providers unfamiliar with a patient’s needs and medical history, inefficient communication and information transfer between healthcare teams, errors in prescribing and administering medications, and limited access to timely care and advice with practical management like managing urinary catheters and nasogastric tubes [15]. Without adequate care and support, unexpected events can result in often burdensome unplanned hospital attendance and admission [20].

Most studies have focused on out-of-hours services provided by primary care teams. Few studies have thoroughly detailed and characterised the use of community out-of-hours palliative care services and explored to what extent these models differ [21]. An understanding of the use of these services is essential to better inform service provision and improve service quality.

Our original systematic review synthesised evidence on the components, outcomes, and economic evaluation of community-based ‘out-of-hours’ care for patients near the end of life and their families [22]. However, there was not scope to report on service utilisation in detail. With limited resources for out-of-hours care, we considered it vital to better understand the evidence on patterns of service utilisation out-of-hours. Therefore, we aimed–in this additional analysis ‐ to synthesise evidence on the use of out-of-hours services by people receiving palliative care in the community; identify patterns of use and provision; and make recommendations for the design of services.

This review aims to answer the following research questions–what is the evidence on:

1. When patients/families/health care professionals need out-of-hours services (time of contact)?
2. Who is contacting the out-of-hours services (who is contacting)?
3. Is it a telephone call, centre visit or a home visit provided (type of contact)?
4. Who responds to out-of-hours calls?

## Methods

This review is an additional analysis of a recently completed systematic review that examined the components, outcomes, and economic evaluation of community-based ‘out-of-hours’ care for patients near the end of life and their families [22].

**Fig 1:**
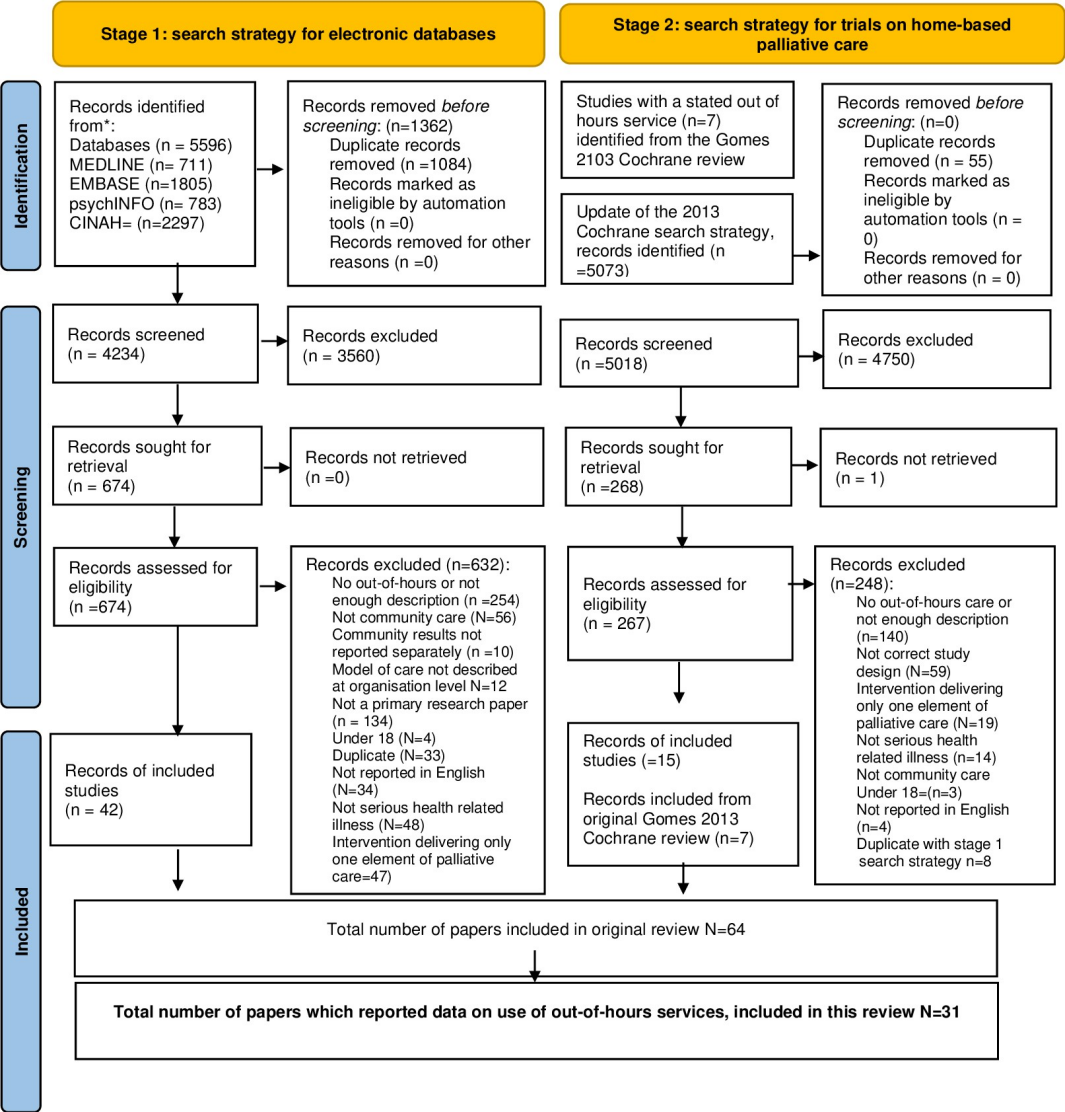
PRISMA flowchart for the original systematic review [22].

The original review was reported following PRISMA guidelines (Fig 1). It was a mixed method systematic narrative review of quantitative and qualitative research studies. A two-stage search strategy was used: first a search of four electronic databases MEDLINE, EMBASE, PsycINFO and CINAHL between 1^st^ January 1990 to 1^st^ August 2022 was undertaken, and then a second search strategy involved identification of trials on home-based out-of-hours palliative care included in the Cochrane review of home-based palliative care [6]. Further details, including MeSH and keyword terms, inclusion and exclusion criteria and quality assessment, are published elsewhere [22].

This study extends the original review by examining in detail the out-of-hours service use (time of contact, who is contacting, type of contact, and who responds to out-of-hours calls), as reported in the included studies on community-based out-of-hours care. Pask et al’s adaptation for palliative care of Bronfenbrenner’s Ecological Systems Theory underpinned both this study and the original review to guide the analysis and interpretation [4]. This adapted theory highlights the many layers that build complexity for patients and families living with advanced illness, including linkage with individual context and environmental factors, such as service-/system-level provision of palliative care that impact the use of out-of-hours services.

In the original review, 64 papers relating to 54 studies met eligibility criteria (from the 9,258 titles screened). This additional analysis involved identification of studies included in the original review reporting data on the use of out-of-hours and other services, and exclusion of those with no data on our additional questions. Data on service use were extracted and tabulated as shown in Table 1, and analysed further to explore patterns of service use and provision [23].

**Table 1:** Studies included for additional analysis: First author, date, country and data included on provision of out of hours care.

First author, publication date and country.	Study title	Data included on out of hours care	Design and content	Reported association with positive outcomes?
Adam (2014) [24] Scotland	Why do patients with cancer access out-of-hours primary care? A retrospective study.	Type of contact	Retrospective review of anonymous case records. 950 contacts OOH. Descriptive study–reason for contact.	Outcomes not reported (N/A). Of those who contacted it was because of a symptom 262/852. Most frequent symptoms were pain.
Ahlner-Elmqvist et al (2004) [25] Sweden	Place of death: hospital-based advanced home care versus conventional care	Number & background of HCPs	Prospective non randomised study	Yes. Place of death: significantly more patients died at home in the AHC group (45%) compared with the CC group (15%). Death at home for the intervention group was associated with living together with someone.
Aranda (2001) [26] Australia	Inpatient hospice triage of ‘after-hours’ calls to a community palliative care service	Time of contact Type of contact	Retrospective review of use of OOH service (n = 244) 70% face to face, 30% telephone	Not reported Changes to model of OOH care implemented–but not what they were
Aristides, M. and A. Shiell (1993) [27] Australia	The effects on hospital use and costs of a domiciliary palliative care nursing service.	Number & background of HCPs	Costs collected including staff time, consumables, overheads and capital. Average number of inpatient days for patients admitted to hospital. Number of admissions to hospital.	Yes. Hospital costs. No statistical difference (Reduction in hospital admissions and lower hospital costs not fulfilled. Increase in overall costs.)
Baird-Bower (2016) [28] Australia	Help is just a phone call away: after-hours support for palliative care patients wishing to die at home.	Time of contact Who is contacting	Retrospective analysis of calls to the telephone line combined with referral and mortality data. It was estimated that for every 100 patients that called the after hours service 28 ED presentations were potentially avoided	Yes. Ambulance or ED visit. Patients who engaged with the after hours service were less likely to contact the ambulance service compared with patients who had never called after hours. Patients who engaged with the service were less likely to present at an ED than patients who had never called after hours. Of patients who wanted to die at home, patients who died at home were more likely to use the after hours service than patients who were admitted to an institutional setting.
Baldry, C. and S. Balmer (2000) [29] England	An audit of out-of-hours advice services provided by hospice staff.	Time of contact Who is contacting Type of contact Number & background of HCPs	Retrospective analysis of calls and visits to hospice inpatient unit	N/A. Further education rolled out to health care professionals on the most common reasons for contacting the hospice
Blankenstein, N., et al. (2009) [30] Netherlands	Out-of-hours palliative care provided by GP co-operatives: Availability, content, and effect of transferred information.	Type of contact	Retrospective phone call analysis	Yes. When information was transferred [shared?] [(25%of palliative patients) patients were referred to hospital less often.
Brettell, R., et al. (2018) [31] England	What proportion of patients at the end of life contact out-of-hours primary care? A data linkage study in Oxfordshire	Time of contact Type of contact	Population-based study linking a database of patient contacts with OOH primary care (n = 102877) with the register of all deaths within Oxfordshire. Access to the service is via NHS111.	N/A OOH services see around one third of all patients who die in a population
Buck, J., et al. (2018) [32] England	Persistent inequalities in Hospice at Home provision	Who is contacting Number & background of HCPs	Retrospective case note analysis (321 patients)	N/A. Twice as many night care episodes were requested (n = 1237) as were provided (n = 613). More staff taken on.
Butler (2013) [33] England	Setting up a new evidence-based hospice-at-home service in England	Number & background of HCPs	Quasi experimental multi centred controlled evaluation	Yes. Preferred place of death. In the control group 61.9% achieved their preferred place of death compared to 63% in the intervention group.
Campbell et al (2005) [34] UK	Introducing ‘Palcall’: an innovative out-of-hours telephone service led by hospice nurses	Time of contact Who is contacting Number & background of HCPs	Practice development report	N/A. Describes number of patients registered and times and purpose of calls.
Carlebach (2010) [35] UK	A review of an out-of-hours telephone support service for palliative care patients and their families	Who is contacting	Evaluation of an out-of-hours service created for palliative care patients in a primary care trust	N/A. Service use reported (52% of calls from carers, 17% from district nurses, 7% from the patient)
Carr et al (2013) [36] USA	PAL-MED CONNECT: A Telephone Consultation Hotline for Palliative Medicine Questions	Who is contacting Number & background of HCPs	Retrospective descriptive of 498 calls received over 2.5 years	N/A.
Dhiliwal et al (2015) [37] India	Impact of Specialist Home-Based Palliative Care Services in a Tertiary Oncology Set Up: A Prospective Non-Randomized Observational Study	Number & background of HCPs	Prospective cohort study? (not clearly stated)	Yes. Psychological care, out-of-hours care, social care, place of death, bereavement support. All patients receiving specialist home care had good relief of physical symptoms. Of the 42.68% who received home based bereavement care, 91.66% had good bereavement outcomes.
Doré 2018 [38] UK	Community palliative medicine out-of-hours needs and the 7-day week: a service evaluation.	Time of contact Type of contact	Analysis of data from an out-of-hours general practice cooperative telephone calls and visits by general practitioners over one year.	N/A
Elfrink et al (2002) [39] Netherlands	Problem solving by telephone in palliative care: use of a predetermined assessment tool within a program of home care technology	Who is contacting	Retrospective evaluation of telephone service	N/A. Telephone service and use of the PAT made it possible to solve 97% problems without admission
Grande et al (2000) [40] UK	A randomized controlled trial of a hospital at home service for the terminally ill	Number & background of HCPs	Randomised controlled trial	Yes. Place of death 1. patients who were admitted to CHAH had significantly longer survival from referral to death compared with patients allocated to CHAH but not admitted to the service (median 16 and 8 days, respectively, Z = 3.005, P = 0.003). 2. Patients who spent time at home during their final 2 weeks had shorter survival from referral than those who did not, whether control or CHAH group (median 10 and 17 days, respectively, Z = 2.849, P = 0.004). Among patients who spent time at home during the final 2 weeks of life, the CHAH and control group did not differ in terms of cancer prevalence, proportion living alone, age, sex or survival from referral. 3. The control and CHAH group did not differ significantly in the proportion who spent time at home during their final 2 weeks (77% versus 82%; χ2 = 0.557, df = 1, P = 0.455). 4. In general, control group was rated higher psychological distress and symptoms from GPs, nurses and informal carers
Jiang et al (2012) [21] USA	A Descriptive, Retrospective Study of After-hours Calls in Hospice and Palliative Care	Time of contact Who is contacting	Retrospective descriptive study (timing of calls, reasons for call and predominant nursing intervention offered)	N/A
Keall 2020 [41] Australia	Extended-hours palliative care service with a hospital-avoidance and enhanced-care approach: report of a quality improvement project.	Time of contact Who is contacting	Data collected during service improvement pilot of extended hours palliative care service on admissions (to palliative care unit), nurse’s assessment of whether acute hospital admission avoided and telephone call log.	Yes. Outcomes: staying at home or preferred place of care, avoiding acute hospitalisation. Extended hours palliative care service, compared with usual care, showed an almost 50% decrease in acute hospitalisation, nearly doubled after-hours palliative care unit admission and 17% increase in patients staying in their own home.
Klarare et al (2017) [42] Sweden	Experiences of security and continuity of care: Patients’ and families’ narratives about the work of specialized palliative home care teams	Number & background of HCPs	Qualitative study -accounts of episodes of out-of-hours care	N/A
Kristianson et al (2004) [43] Australia	Evaluation of a night respite community palliative care service	Number & background of HCPs	Action research with some cost data collected, also number of night respite shifts required, place of death	Yes. There is evidence that patients who would have been transferred to an inpatient setting for end-of-life care were able to die at home with the support of the night respite service. Families were extremely appreciative of the service and a limited cost comparison suggested costs were lower than if patients had been transferred to hospital and/or inpatient hospice care.
Marshall et al (2008) [44] Canada	Enhancing family physician capacity to deliver quality palliative home care: An end-of-life, shared-care model	Who is contacting Number & background of HCPs	Description of programme development	N/A
Masso et al (2007) [45] Australia	GAPS revisited: follow up evaluation of an Australian rural palliative care service	Type of contact	Service audit, descriptive	N/A
Middleton-Green et al (2016) [46] UK	‘A Friend in the Corner’: supporting people at home in the last year of life via telephone and video consultation—an evaluation	Time of contact	Evaluation of ‘Gold Line’, 24/7 nurse-led telephone and video consultation support service for patients in last year of life	No, evaluation was descriptive. However, claim ‘98.5% of calls resulted in patients remaining in their place of residence’.
Phillips et al (2008) [47] Australia	Supporting patients and their caregiver’s after-hours at the end of life: the role of telephone support.	Time of contact Who is contacting	Evaluation (documentary analysis and qualitative interviews)	No, but concluded that ‘acceptable palliative care advice can be provided by generalist nurses in a cost efficient manner’ and a range of initiatives set up as a result
Plummer et al (2006) [48] UK	Reviewing a new model for delivering short-term specialist palliative care at home	Number & background of HCPs	Service evaluation (audit and questionnaire)	N/A
Riolf 2014 [49] Italy	Effectiveness of palliative home-care services in reducing hospital admissions and determinants of hospitalization for terminally ill patients followed up by a palliative home-care team: A retrospective cohort study	Number & background of HCPs	Retrospective controlled cohort and case control study	Yes. Reducing hospital admissions: patients enrolled for the palliative care programme were more likely to die at home and fewer hospital stays and shorter stays in their last two months of life compared with those who were not taken onto the programme.
Rosenquist (1999) [50] Sweden	Optimizing hospital-based home care for dying cancer patients: a population-based study.	Number & background of HCPs	Retrospective document analysis looking at place of death and hospital-based home care (and reasons for not having hospital based home care)	Outcomes not recorded but ‘results showed that a higher home death rate could be achieved if effective hospital based home care were offered (and accepted)’
Shabnam et al (2018) [51] Bangladesh	24/7 palliative care telephone consultation service in Bangladesh: A descriptive mixed method study ‐ They know that we are with them	Who is contacting Number & background of HCPs	Descriptive analysis of telephone calls and group interview with five palliative care physicians.	N/A
Wilkes et al (2004) [52] Australia	Evaluation of an after-hours telephone support service for rural palliative care patients and their families: A pilot study	Number & background of HCPs	Descriptive evaluation (audit of calls, text analysis of reflective journals, questionnaire and interviews)	N/A
Worth et al. (2006) [1] Scotland	Out-of-hours palliative care: A qualitative study of cancer patients, carers and professionals	Type of contact	Qualitative study: interviews with patients who had used out-of-hours services and/or their carers; focus groups with patients and carers and telephone interviews with their general practitioners and other professionals	N/A

Key:

Time of contact = When do patients/families/health care professionals use out-of-hours services?

Who is contacting = Who is contacting the out-of-hours services?

Type of contact = Is it a telephone call, centre visit or a home visit?

Number & background of healthcare professionals (HCPs) = How many healthcare professionals are on duty? And what are their backgrounds?

## Results

From 64 papers included in the original review [22], 31 reported data on use of out-of-hours services from ten different countries (Table 1). 33 provided no information on the use of services, and were therefore excluded from the analysis. Over half the studies reported on the number and background of healthcare professionals on duty (n = 17) (S4 Table), with fewer reporting on who contacted the service (n = 12) (S2 Table), time of contact (n = 10) (S1 Table), and the type of contact (n = 8) (S3 Table). Detailed analysis on patterns of service use is reported in supplementary material (S1–S4 Tables). The papers report a range of methods, and few report outcomes, and only four report outcomes associated with extended or out-of-hours service provision [28, 30, 41, 43].

### Overview of included studies

#### 1. When do patients/families/health care professionals need out-of-hours services?

Ten papers provided data about the time of contact. Five papers reported the highest use of out-of-hours services as in the evenings, particularly between 5pm-12pm [21, 26, 28, 29, 47]. Three of these papers referred to specific out-of-hours services and two papers referred to 24/7 services. Jiang et al (2012) reported that 31.1% (n = 1,381) of calls occurred at the weekend (the second most busy time out-of-hours in this study) [21]. However, Dore (2018) reported that for an out-of-hours GP service, weekends were the busiest time for interactions with palliative patients, and that the distribution of contacts over the weekend was evenly spread from midnight to 9pm [38]. Keall (2023) reported that for an extended hours community palliative care service, peak times were at weekends between 10am and 1pm and Saturdays were busier than Sundays [41].

Three studies based on 24/7 services showed that out-of-hours calls made up a substantial proportion of all calls to the services [29, 41, 46]. Middleton-Green et al (2016) report that the majority of the calls were made out-of-hours (69%, n = 3523), whilst Baldry and Palmer (2000) report that nearly half of all calls were out-of-hours calls (48%, n = 211).

#### 2. Who is contacting the out-of-hours services?

Twelve papers provided results for who contacted out-of-hours services, and this included patients, families, and healthcare professionals. Four papers referred to specific out-of-hours services and eight papers referred to 24/7 services. Family members and carers were the most frequent contacters of out-of-hours services. Seven studies out of the eleven which compared the different groups contacting the out-of-hours services showed that this group were the most frequent [28, 29, 34, 35, 41, 47, 51]. Six studies showed more than 60% of all calls were from family members or carers, with two studies showing as much as 80% of all contacts being from family members or carers [47, 51]. Furthermore, three studies showed that family members and patients together formed the majority of callers attributing this group to 57.1%, 59% and 79% of all calls, respectively [21, 41, 44].

Healthcare professionals were the most frequent contacts in one study where, out of all incoming and outgoing calls, 56% (n = 838) of calls were with healthcare staff including generalists and 42% (n = 636) with family and carers [32]. In a further study of a palliative medicine telephone hotline for generalists caring for community patients, most calls (43%) came from physicians, with 22% from registered nurses, 15% from nurse practitioners and 6% from pharmacists [36].

#### 3. Is it a telephone call, centre visit or a home visit?

Eight papers provided results on the type of contact reported following a call to out-of-hours services: follow up by telephone, or a home visit, or the patient visiting a service centre out-of-hours.

Of the three papers which reported on the proportion of type of follow up after a phone call, home visits were the most common in two studies. In a retrospective study of all palliative care telephone calls to an out-of-hours GP co-operative over a one year period (2005 to 2006) 53% of calls (n = 551) resulted in a home visit and just 0.9% (n = 9) in a centre visit [30]. In two other retrospective studies of community palliative care services (analysing data collected in 1996/97 and 2003 to 2007), 40% (n = 251) and 52.1% (n = 325) of calls respectively resulted in home visits [26, 45]. One paper analysed patient interactions with an out-of-hours GP service over one year. Palliative care made up 11.4% of the total out-of-hours GP home visits for this service, or161 interactions with palliative patients. Of these, 114 were home visits [38].

Of the four papers which reported on type of subsequent contact, by proportion of all contacts, home visits were the most common in three studies. In a retrospective study in a primary care out-of-hours department, out of 950 individual patient consultations, 71.3% were home visits, 22.3% were telephone consultations and 6.4% were centre visits [24]. In addition, in an out-of-hours qualitative study 78% (n = 25) of contacts were home visits. In a 24/7 specialist advisory service 84% (n = 178) were telephone calls [29].

One study showed differences in types of consultation in patients within 30 days of death and patients not within 30 days of death [31]. In patients within 30 days of death (n = 2661), home visits were the most common at 55.8% of contacts, 4.2% were centre visits and 39.9% were telephone calls. In patients not within 30 days of death (n = 100,216), centre visits were the most common at 55.8%, 9.7% of contacts were home visits and 34.3% were telephone calls. In a paper that analysed out-of-hours contacts with GPs, 55% of patients who received home visits were judged to be within 48 hours of death [38] No indication was given of an association between home visits and outcomes for patients.

#### 4. How many healthcare professionals are on duty? And what are their backgrounds?

Of the17 studies which reported the backgrounds of the staff delivering care out of hours, 14 studies reported staff from specialist palliative care services and two reported integrated specialist and generalist services. Eight studies reported services led by specialist nurses in palliative care and did not report whether there was access to a specialist medical doctor/consultant [26, 31, 32, 39, 42, 47, 49, 51]. Six studies had at least one specialist medical doctor as part of the service team [24, 35, 36, 43, 48, 50] and thirteen studies with at least one specialist nurse [25, 27, 32–34, 36, 37, 40, 42, 44, 48, 50, 52]. No data were collected on outcomes for patients in relation to professional background of the service team.

#### 5. Reported outcomes

Although a number of papers reported outcomes associated with the service described they did not specify whether these were associated with the out-of-hours component of the service. However, there were four papers which reported outcomes for services set up specifically to provide out-of-hours or extended hours support for palliative patients [28, 30, 41, 43]. Baird-Bower (2016) reported that patients who engaged with the after hours telephone service were less likely to contact the ambulance service or present at the emergency department than patient who had never called out of hours. An analysis of shared records in out-of-hours GP co-operatives showed that when information was shared patients were referred to hospital less often [30]. An extended hours palliative care service, compared with usual care, showed an almost 50% decrease in acute hospitalisation and 17% increase in patients staying in their own home [41]. An evaluation of a night respite community palliative care service suggested that there is evidenced that the service enabled patients to die at home and that the healthcare costs were lower for these patients [43].

## Discussion

This review sought to understand the use of out-of-hours palliative care services in the community, to inform the design of services. The findings demonstrate that overall, out-of-hours palliative care services were most used in the evenings, especially on weekdays. This finding was particularly evidenced in studies of 24/7-hour services (which include in-hours service) emphasizing the extra demand during evenings, the requirement for out-of-hours services, and the implications for planning ahead by ‘in-hours’ services. The review findings show that family members and carers were the most frequent callers of out-of-hour services, as indicated by more than half the included studies which reported on caller identity. This evidence does not, however, tell us about those who do not access out-of-hours services despite need, or who are put off. Previous research shows that some patients and carers find out-of-hours care difficult to access, for example not knowing who to call, or finding the triage process challenging. Patients and carers can also be hesitant to call, worrying about the legitimacy of their need [1].

Our original systematic review of models of out-of-hours services [22] found improved patient outcomes for community out-of- hours services provided 24/7 by specialist palliative care services offering both hands-on clinical care and advisory care. However, resources are limited, notably workforce shortages, meaning that this model is not always feasible. Therefore, understanding more about the evidence on patterns of service use is important. The time of greatest use is an important aspect to consider in the design of services. It is interesting that evenings were apparently usually busier than weekends (as shown in studies from Australia, USA and UK). We know from other research that symptom control, in particular pain control is a main reason for contacting services out-of-hours [24], but it would be helpful to record systematically the reasons behind the calls when collecting out-of-hours data in future. The studies which reported length of time of contact were all focused on telephone calls, with none specifically on home visits. Further research into the frequency of home visits would help planning and allocation of time for potential unscheduled visits. In light of this, commissioners and service providers should ensure that adequate telephone services (for advice and to assess whether a visit is needed) are available and sufficient at these times. Moreover, other studies have advocated offering proactive evening telephone calls, especially for frequent service users or for patients with persistent symptoms, with the aim of minimizing problems and reducing the need for home visits during the night [21, 26].

Out-of-hours services for palliative patients should be designed with careful consideration of the needs of family carers. Services should provide clear contact details and provide information on how to use the services appropriately, reducing anxiety for patients and carers.

In relation to our four research questions, some patterns of use were less clear. The type of contact was highly varied between studies, not always reported, and it is therefore difficult to draw any conclusions. Some studies reported only the type of contact following the first call, whereas others reported on the type of contact overall. Furthermore, no studies reported on whether home/telephone visits met the patient’s need or not, making it difficult to make meaningful comparisons across evidence. Future studies looking at patient outcomes and patient experience following out-of-hours contact would be beneficial to assess the meeting of patients’ needs and to provide more context on service use. The number and ratio of different healthcare professionals providing out-of-hours services varied among the included studies and was difficult to elucidate. An interesting finding was that a number of studies reported no doctor as part of the palliative care team. Doctors have an important role in the coordination of care, continuity of care and accessing of prescriptions [53], although nurse prescriber roles may assist with this. The implications of not having access to a doctor on service provision and quality of care is not known.

Finally, most of the studies were descriptive and the evidence presented in terms of outcomes for patients was limited. Where positive outcomes were reported, there was no detailed information on whether these were associated with, for example, the professional backgrounds of the staff providing the service, or whether the outcomes varied according to whether the interaction was a telephone call or a home visit.

### Strengths and limitations

This review examined the available international data on the use of out-of-hours community palliative care services and makes clear recommendations for service design. Provision of community and primary care varies widely depending on health care systems, but it is possible to see that there are similar patterns across countries (such as the times when patients and families need services), and recommendations such as involving patients and families in service design hold true everywhere. Results of this study should be interpreted in the light of several limitations. First, although studies were carefully and systematically selected, the evidence was of varying quality, and differently collected and described, which made the synthesis of some findings difficult. Secondly, the majority of the studies included in this review were not recent and were pre-COVID-19. A UK based study on end-of-life care showed services adapted rapidly to the pandemic: there was an increased need and provision of care, particularly in face to face visits by community nurses with patients choosing to remain at home rather than being admitted to hospital [54]. There were also changes to work patterns due to symptom management and staff shortages. Therefore, this should be taken into consideration in the interpretation of findings.

## Conclusion

This review provides a synthesis of the current evidence available on service utilisation out-of-hours by people receiving palliative care in the community. The review identified that family carers are the most likely to contact out-of-hours service and therefore services should be designed with consideration to the specific needs of family carers. Out-of-hours services are provided by a range of services and provision is multi-professional, it is vital to ensure these services are well equipped with enhanced resources in the evenings to ensure out-of-hours services are well equipped. This review also highlights areas for further research in this field, and for the need for more comprehensive and systematic collection of data on out-of-hours service use. The findings from this review have demonstrated the need for out-of-hours service and provide valuable recommendations to commissioners and service providers. These can be used to improve the provision and delivery of current community-based out-of-hours palliative care services and facilitate care towards the end of life.

## Recommendations

When commissioning and designing out-of-hours palliative care services, consideration should be given to:

- Ensure services increase provision of out-of-hours services between 5pm and midnight to reflect the increased use at these times.
- Ensure that family and carers are provided with clear contact details for out-of hours support.
- Ensure patient records can be easily accessed by health professionals responding to calls, making the triage process easier.
- Listen to patients, family and carers in the design of out-of-hours services, including telephone services.
- Collect data systematically on out-of-hours-service use and on outcomes for patients who use the service.

## Supporting information

S1 Table Summary of studies reporting the time of contact of out-of-hours services. (DOCX)

S2 Table Summary of studies reporting who is contacting out-of-hours services. (DOCX)

S3 Table Summary of studies reporting the type of contact. (DOCX)

S4 Table Summary of studies reporting the number & background of HCPs. (DOCX)

S1 Checklist PRISMA checklist. (DOCX)
