# Supplementary material for: Understanding patient and family utilisation of community-based palliative care services out-of-hours: Additional analysis of systematic review evidence using narrative synthesis
Source: PLoS One. 2024 Feb 21;19(2):e0296405. doi: 10.1371/journal.pone.0296405 (PMC10880966; doi:10.1371/journal.pone.0296405)
Supplement: S1 Table — (DOCX) [file pone.0296405.s001.docx]

**Supplementary Table 1:** Summary of studies reporting the time of contact of out-of-hours services

| Paper | Time of contact |
| --- | --- |
| Aranda (2001) | Predominantly between 5pm and 11pm (62.5%). A further 8.3% were made between 7am and 8.30am. Calls evenly distributed across the year with a monthly average of 26.2 calls (range 9-42). |
| Baird-Bower (2016) | predominant times as between 4pm and 11pm with many between especially between 4-5pm (19.3%). predominant time of contact over the weekend and public holidays is between 9 am and 10 am. Weekday calls made up 46% of out-of-hours calls whereas weekend calls made up 52% and public holidays made up 2% |
| Baldry, C. and S. Balmer (2000 ) | 52% calls between 9am and 5pm, 48% calls were out-of-hours. 27% out-of-hours calls between 5pm and 10pm and 10% between 10pm and 9am on weekdays/nights |
| Brettell, R., et al. (2018) | Contacts within 30 days of death, overnight 00:00–07:59 hours= 22.6%, Evening 18:30–23:59 hours= 29.4%, Daytime 08:00–18:29 hours = 48.0%. Contacts not within 30 days of death Overnight 00:00 –07:59 hours= 15.5%, Evening 18:30–23:59 hours= 37.8%, Daytime 08:00–18:29 hours = 46.7%. |
| Campbell et al (2005 ) | On weekends and bank holidays - 44% of calls between 9am - 6pm. On weekdays 41% of calls between 6pm and midnight and 15% of calls Midnight - 9am. |
| Phillips et al (2008) | Predominant time between 6 pm and midnight. (78%) |
| Jiang et al (2012) | Predominant time between 5pm and 12am (73.1%). 26.9% calls between 12AM and 8AM. 68.5% calls were made after hours on weeknights. Nearly one-third (31.1%) of calls took place on weekend. 6.3% were made on holidays |
| Middleton-Green et al (2016) | 69% of calls were out-of-hours |
| Doré, M and Willis, D (2018) | During weekdays, 40% of the workload was from 17:00 to 21:00, 25% from 21:00 to 00:00, and 35% from 00:00 to 09:00. During the weekend, 26% of the workload was from 09:00 to 12:00, 26% from 12:00 to 17:00, 22% from 17:00 to 21:00, 8% from 21:00 to 00:00, and 26% from 00:00 09:00. |
| Keall, R and Lovell, M (2023) | Peak times were at weekends 10:00–13:00, and Saturdays were busier than Sundays. |
