# Supplementary material for: Understanding patient and family utilisation of community-based palliative care services out-of-hours: Additional analysis of systematic review evidence using narrative synthesis
Source: PLoS One. 2024 Feb 21;19(2):e0296405. doi: 10.1371/journal.pone.0296405 (PMC10880966; doi:10.1371/journal.pone.0296405)
Supplement: S2 Table — (DOCX) [file pone.0296405.s002.docx]

**Table 2** Summary of studies reporting who is contacting out-of-hours services

| Paper | Family/carers % | Patients % | HCPs % |
| --- | --- | --- | --- |
| Baird-Bower (2016) | 77 | 6 | 16 |
| Baldry and Balmer (2000) | 62 | 10 | 28 |
| Buck et al (2018) | 7 | n/a | 21 |
| Carlebach (2010) | 52 | 7 | 17 |
| Carr et al (2013)  HCP only hotline | 0 | 0 | 43% physicians  22% reg. nurses  15% nurse practitioners  6% pharmacist |
| Jiang et al (2012) | 57.1% patient or family | 57.1% patient or family | 28.8% |
| Elfrink et al (2002) | 73 in 1997 (family and patients)  47 in 1998  28 in 1999 | n/a | 12 in 1997 (district nurse)  35 in 1998  48 in 1999 |
| Campbell et al (2005) | 64 | 22 | 14 |
| Marshall et al (2008) | 59 (family and patients) | 59 | 35 |
| Phillips et al (2008) | 80 | 20 | 0 |
| Shabnam et al (2018) | 80 | n/a | n/a |
| Keall, R and Lovell, M (2023) | 61 | 18 | 21 |
