# Supplementary material for: Understanding patient and family utilisation of community-based palliative care services out-of-hours: Additional analysis of systematic review evidence using narrative synthesis
Source: PLoS One. 2024 Feb 21;19(2):e0296405. doi: 10.1371/journal.pone.0296405 (PMC10880966; doi:10.1371/journal.pone.0296405)
Supplement: S3 Table — (DOCX) [file pone.0296405.s003.docx]

**Table 3** Summary of studies reporting the type of contact

| Paper | Type of contact |
| --- | --- |
| Adam (2014) | Of all consultations, 71.3% home visits, 22.3% telephone consultations, 6.4% centre visits. |
| Aranda (2001) | Of 629 calls, 40% (251) resulted in home visits |
| Baldry, C. and S. Balmer (2000) | Of 211 out-of-hours calls/visits, 178 were telephone calls |
| Blankenstein, N., et al. (2009) | 46% telephone consultation, 0.9% centre visit, 53% home visit |
| Worth et al. (2006) | Most home visits - of 36 patients recruited, 25 patients had received a GP visit, 6 a district nurse visit, 5 had attended the out-of-hours centre, and 3 received telephone advice only |
| Brettell, R., et al. (2018) | Most home and telephone in patients within 30 days of death: (n=2661), Home visit = 55.8%. Base assessment = 4.2%, telephone 39.9%.  Most phone and centre in patients not within 30 days of death: (n= 100 216), Home visit = 9.7%, base assessment = 55.8%, telephone 34.3% |
| Masso et al (2007) | Of all calls, 52.1% resulted in home visits |
| Doré, M and Willis, D (2018) | 161 palliative patient interactions over one year, of which 114 were home visits |
