# Supplementary material for: Understanding patient and family utilisation of community-based palliative care services out-of-hours: Additional analysis of systematic review evidence using narrative synthesis
Source: PLoS One. 2024 Feb 21;19(2):e0296405. doi: 10.1371/journal.pone.0296405 (PMC10880966; doi:10.1371/journal.pone.0296405)
Supplement: S4 Table — (DOCX) [file pone.0296405.s004.docx]

**Table 4** Summary of studies reporting the number & background of HCPs

| Paper | SPC doctors | SPC nurses | Other doctors/GPs | District and community nurses | HCAs/care aides | Other |
| --- | --- | --- | --- | --- | --- | --- |
| Aristides, M. and A. Shiell (1993) | NA | 18 | NA | NA | NA | NA |
| Buck et al (2018) | NA | 6 hospice nurses  1 specialist nurse manager | NA | 2 bank nurses | 14 | 1 therapist  2 admin assistants |
| Butler 2013 (join with Holdsworth, and Gage 2015 | NA | Community clinical nurse specialist | NA | District nurses | 16.8 whole time equivalent HCAs | NA |
| Carr et al. 2013 | 18 palliative physicians | 7 hospice nurses | NA | NA | NA | NA |
| Dhiliwal et al 2015 | 3 | 1 | NA | NA | NA | 1 social worker |
| Campbell et al 2005 | NA | 2 hospice nurses | 1 GP | 1 district nurse | NA | NA |
| Kristianson et al 2004 | NA | NA | NA | NA | 15 | NA |
| Marshall et al 2008 | 1 pall med physician | 1 specialist nurse | 21 GPs | 6 community nurses | Bereavement counsellor  Psychosocial-spiritual advisor  Case manager | NA |
| Plummer et al 2006 | NA | 4 | NA | NA | NA | NA |
| Rosenquist 1999 | NA | At least 3 nurses (on shift) | NA | NA | NA | NA |
| Shabnam et al 2018 | 5 | NA | NA | NA | NA | NA |
| Klarare et al 2017 | NA | 30 HCPs doesn’t specify | NA | NA | NA | NA |
| Ahlner-Elmqvist et al 2004 | 1 | 9 | NA | NA | NA | 1 physio  1 social worker  1 secretary |
| Grande et al 2000 | NA | 6 | NA | additional agency nurses (not specified) | 2 | 1 nurse coordinator |
| Wilkes et al 2004 | NA | 21 (mix of specialist and non-specialist nurses) | NA | NA | NA | NA |
| Riolf 2014 | 2 palliative care physicians | NA | NA | 30 non-specialist nurses | NA | NA |
